# Complete chloroplast genome of a novel chlorophyll-deficient mutant (*clm*) in sweetpotato (*Ipomoea batatas* L.)

**DOI:** 10.1080/23802359.2020.1869616

**Published:** 2021-03-18

**Authors:** Hongda Zou, Xiongjian Zhang, Jingyi Chen, Zhangying Wang, Lifei Huang, Boping Fang

**Affiliations:** Guangdong Provincial Key Laboratory of Crops Genetics and Improvement, Crops Research Institute, Guangdong Academy of Agricultural Sciences, Guangzhou, China

**Keywords:** *Ipomoea batatas*, chloroplast genome, chlorophyll-deficient mutant

## Abstract

The complete chloroplast genome of a novel chlorophyll-deficient mutant (*clm*) and its wild type (WT) in sweetpotato (*Ipomoea batatas* L.) was sequenced. The complete chloroplast genome of *clm* and WT was 161,393 bp and 161,429 bp in length, containing a large single copy (LSC) region of 87,561 bp and 87,597 bp, respectively, a small single copy (SSC) region with the same length of 30,890 bp and a pair of inverted repeat regions (IRs) with the same length of 12,052 bp. Both of them contained 132 genes including 87 protein-coding sequences, 37 tRNA, and eight rRNA. Comparing to the WT, four SNPs and three INDELs were detected and only one INDEL in the exon affecting the translation of *rpoA* gene. Phylogenetic analysis showed that *clm* and WT were closely related to *Ipomoea tabascana*. The complete chloroplast genome of *clm* and its WT will play a role in understanding the molecular mechanism of chlorophyll deficiency and developing molecular markers in sweetpotato.

The sweetpotato (*Ipomoea batatas* L.) is generally vegetatively propagated by means of roots or cuttings. The sports (vegetative mutations) are fairly universal in sweetpotato cultivation (Gustafsson and Gadd 2009). As one kind of common mutations in sweetpotato, although the chlorophyll-deficient mutant was found as early as 1952 (Kuwata 1950), its physiological and molecular mechanisms have not been studied in detail. The chlorophyll-deficient mutant is a valuable material for research in chlorophyll biosynthesis, chloroplast development and structure function, photosynthesis mechanism, gene function, as well as ornamental plant breeding (Frank and Chitwood 2016). Here, we first sequenced and characterized the complete chloroplast genome of a novel chlorophyll-deficient mutant (*clm*) and its wild type (WT) in sweetpotato (*Ipomoea batatas* L.), and analyzed their phylogenetic relationship in *Ipomoea* genus.

The fresh leaves of *clm* and WT were collected in Guangzhou, Guangdong Province, China (N23°39′41.36″; E113°44′04.18″). Voucher specimens and DNA were deposited in the Crops Research Institute, Guangdong Academy of Agricultural Sciences (voucher number: CRI20191024). The total genomic DNA of *clm* and WT was extracted using the Plant Genomic DNA Kit (Tiangen, Beijing, China). The whole genome sequencings were conducted by Nanjing Genepioneer Biotechnologies Inc. (Nanjing, China) using Illumina Hiseq 2500 system. The raw reads were filtered by Trimmomatic v0.32 (Bolger et al. 2014) and mapped to Xushu 18 chloroplast genome (Yan et al. 2015) as the reference by Bowtie2 v2.35 (Langmead and Salzberg 2012). The obtained high-quality chloroplast reads were assembled in chloroplast genome by SPAdes v3.10 (Bankevich et al. 2012). The final annotations were performed using GeSeq (Tillich et al. 2017) and Blast search.

The complete chloroplast genome of *clm* (accession number MW122506) and WT (accession number MW122507) was 161,393 bp and 161,429 bp in length, containing a large single copy (LSC) region of 87,561 bp and 87,597 bp, respectively. Both of them had a small single copy (SSC) region with the same length of 30,890 bp and a pair of inverted repeat regions (IRs) with the same length of 12,052 bp. The annotation results showed that both the chloroplast genome of *clm* and WT contained 132 genes, including 87 mRNA, 37 tRNA, and eight rRNA. The structure variation between *clm* and WT chloroplast genome was screened by MAFFT v7.450 (Katoh and Standley 2013) and perl script. Comparing to WT, four SNPs in *ycf1* gene and three INDELs in *rpoA* gene were identified in *clm*. But there was only one INDEL variation affecting gene expression. The rpoA protein was shortened by the alternation in exon locus of *rpoA* gene, which may lead to the phenotype of chlorophyll-deficient.

To figure out the phylogenetic status of *clm* and its WT in *Ipomoea* genus, the maximum-likelihood tree was constructed based on eight complete chloroplast genomes of *Ipomoea* species from GenBank, and *Cressa cretica* and *Cuscuta reflexa* were used as the outgroup. These 12 whole chloroplast genomes were aligned using MAFFT v7.450 (Katoh and Standley 2013) and the maximum-likelihood tree was constructed using RAxML v8.2.10 (Stamatakis 2014) with 1000 bootstrap value (Figure 1). The result of phylogenetic analysis showed that the *clm* mutant and its WT were clustered together, and close to *Ipomoea tabascana*. It was further confirmed that *I. tabascana* could be a recent hybrid between *I. batatas* and *I. trifida*, and there were two *I. batatas* lineages due to chloroplast capture (Muñoz-Rodríguez et al. 2018). The complete chloroplast genome of *clm* and its WT will play an important role in understanding the molecular mechanism of chlorophyll deficiency and developing molecular markers in sweetpotato.

**Figure 1.**
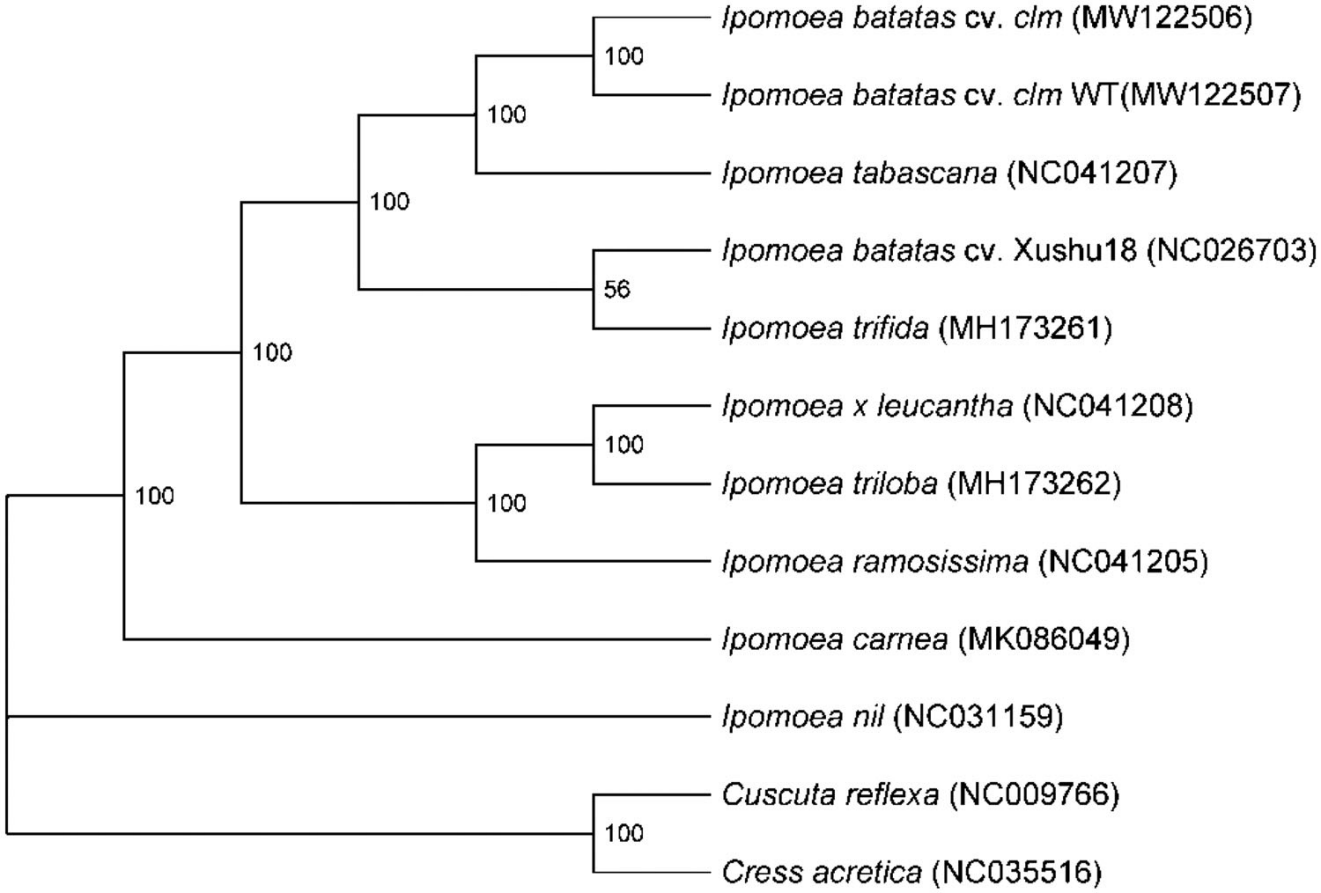
The phylogenetic analysis of the *clm* and its WT in *Ipomoea* genus based on complete chloroplast genome using maximum-likelihood (ML) method.

## Data Availability

The genome sequence data that support the findings of this study are openly available in GenBank of NCBI at (https://www.ncbi.nlm.nih.gov/) under the accession nos. MW122506 and MW122507. The associated BioProject and BioSample numbers are PRJNA684785, and SAMN17069323 and SAMN17069324, respectively.
